# Explainable analytics: understanding causes, correcting errors, and achieving increasingly perfect accuracy from the nature of distinguishable patterns

**DOI:** 10.1038/s41598-022-19650-2

**Published:** 2022-11-01

**Authors:** Hao-Ting Pai, Chung-Chian Hsu

**Affiliations:** 1 Bachelor Program of Big Data Applications in Business, National Pingtung University, Pingtung, Taiwan; 2 Department of Information Management, National Yunlin University of Science and Technology, Douliou, Taiwan; 3 International Graduate School of Artificial Intelligence, National Yunlin University of Science and Technology, Douliou, Taiwan

**Keywords:** Computational science, Computer science, Information technology, Scientific data

## Abstract

In addition to pursuing accurate analytics, it is invaluable to clarify how and why inaccuracy exists. We propose a transparent classification (TC) method. In training, data consist of positive and negative observations. To obtain positive patterns, we find the intersection between each of the two positive observations. The negative patterns are obtained in the same manner. Next, pure positive and pure negative patterns are established by selecting patterns that appear in only one type. In testing, such pure positive and pure negative patterns are used for scoring observations. Next, an observation is classified as positive if its positive score is not zero or if both its positive and negative scores are zero; otherwise, it is classified as negative. By experiment, TC can identify all positive (e.g., malignant) observations at low ratios of training to testing data, e.g., 1:9 using the Breast Cancer Wisconsin (Original) and 3:7 using the Contraceptive Method Choice. Without fine-tuned parameters and random selection, the uncertainty of the methodology is eliminated when using TC. TC can visualize causes, and therefore, prediction errors in a network are traceable and can be corrected. Furthermore, TC shows potential in identifying whether the ground truth is incorrect (e.g., identifying diagnostic errors).

## Introduction

Accurate prediction plays a pivotal role in analytics; however, in reality, researchers usually face the challenge of explaining how and why a prediction is inaccurate^1,2^. According to a survey^3^, outpatient diagnostic errors occur at a rate of 5.08% (approximately 12 million US adults) per year. Even a 1% reduction in errors can save the lives of millions of people. We consider three major types of errors. The first error type, *faults in data*, includes human mistakes or defective instrumentation, from which bad data is produced. Without domain knowledge, this type of fault is difficult to correct. Nevertheless, we should remove inconsistencies, i.e., when observations in the positive class are identical to those in the negative class. In addition, positive and negative observations may have similar patterns that are inextricably interwoven, e.g., people with similar profiles may exhibit different behaviors. Lim et al*.*^4^ showed that the contraceptive method choice (CMC) dataset^5^ is the most difficult to classify, and the minimum error rates are greater than 0.4. The second type of error is related to *mismatches between the data and the methods*. Data, which contain categorical (e.g., country), numerical (e.g., age), or both types of information, place natural constraints on the analysis. For categorical information, only the number of items and the mode are statistically relevant^6^. Therefore, a numerical-orientated method is inherently inadequate for categorical data. Numerical values can be transformed into categorical values by discretization^7^, which is a technique that has been widely applied to knowledge discovery and data mining (KDD) applications^8^. However, bias occurs if categories are not representative of numerical values. The third error is the *big data challenge*, i.e., the complexity of data is determined by the number of rows and features (columns). Particularly, computation tasks increase rapidly with the number of features, which is known as the curse of dimensionality (CoD)^9^. To address CoD, dimension reduction and feature selection methods are utilized to reduce the complexity by extracting information that is practical for classification and cluster analysis. The extraction process, which is a trade-off between efficiency and effectiveness, may involve pruning large amounts of data. There may be pitfalls^10^ in this process, and information related to errors may be missed.

## Results

**Figure 1:**
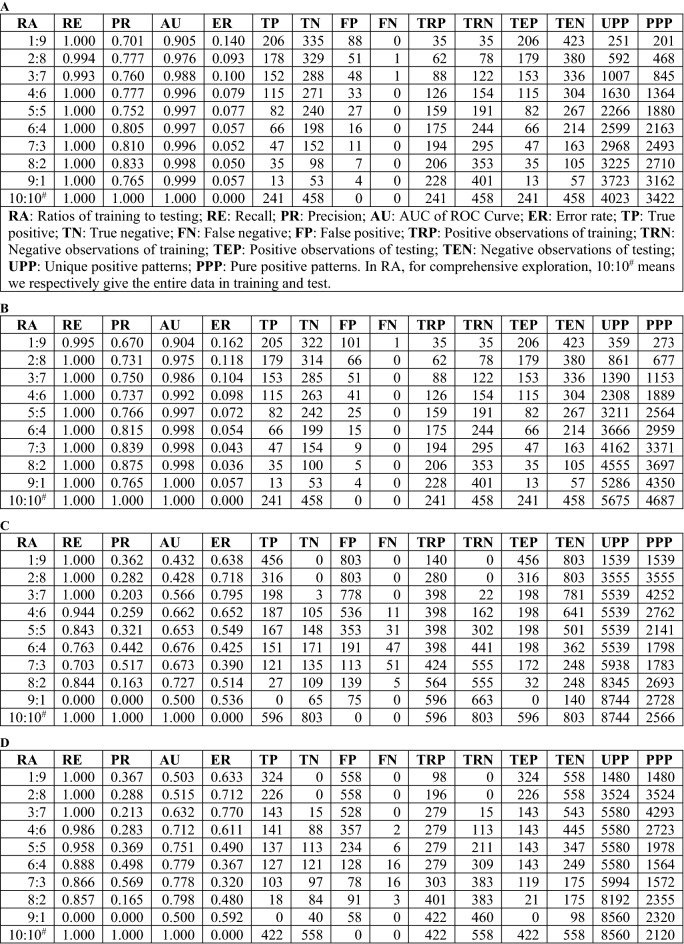
Performance of TC in distinguishing between observations. (**A**, **B**) In Breast Cancer Wisconsin (Original) data set (BCWO), we map class values “malignant” to “1” and “benign” to “0”. In case (**A**), the granularity of discretization is to the first decimal place, e.g., 1.68≈1.6, while in case (**B**) we take an integer for the granularity, e.g., 1.68≈1. (**C**, **D**) In Contraceptive Method Choice data set (CMC), we map class values “1 = No-use” to “1”, “2 = Long-term” to “0”, and “3 = Short-term” to “0”. For (**C**) and (**D**), we set the same granularity as that of (**A**) and (**B**), respectively. For consistency, we remove observations that have identical features but different class labels. The number of observations is thus reduced from 1473 to 1399 in (**C**) and from 1473 to 980 in (**D**).

We conducted experiments with two public real-world datasets: the Breast Cancer Wisconsin (Original) (BCWO) and Contraceptive Method Choice (CMC) datasets, which are available in the UCI Machine Learning Repository^5^. Figure 1A,B show the results on the BCWO dataset, and Fig. 1C,D show the results on the CMC dataset. In Fig. 1A, perfect recall (i.e., a recall of 1.0) is achieved at the lowest ratio (i.e., 1:9) and 7 other ratios using the TC method. This means that this method is not only accurate for small amounts of data but is also stable when the amount of data increases. One error at the ratio of 2:8 and one error at the ratio of 3:7 occur because the positive observation *PO*_223_ is predicted as a negative observation. At the ratio of 4:6, *PO*_223_ is part of the training data but is not used in the testing data. Upon further exploration, at a ratio of 10:10^#^, other than *PO*_223_, the observations are irrelevant to the PPPs. Indeed, *PO*_223_ is related to the PPs, which is also relevant to the NOs. PPPs can eliminate the influence of *PO*_223_ on other observations. Novel observations are regarded as positive observations if they are unrelated to both PPPs and PNPs. Some NOs are predicted to be positive. As a result, a lower performance in terms of PR is observed when using the TC method. With an increased number of training observations, sufficient PPs or NPs may be obtained to provide accurate results and their causes, e.g., at the ratio of 1:9, *PO*_627_ has a novelty score of 70 by Rule 3; at the ratio of 2:8, *PO*_627_ contains 3 patterns in PPPs, namely, ['8|0.8'], ['6|1.5', '8|0.8'], and ['1|0.9', '8|0.8'], and has a positive score of 16 by Rule 1. Figure 1B shows that the suitable granularity of discretization provides a broad overview of how to discover more information. At a ratio of 1:9, one error, which is also caused by *PO*_223_, results when using the TC method. At a ratio of 2:8, *PO*_223_ has 1 pattern ['3|0.0', '5|0.0'] in the PPPs and is related to *PO*_33_ and *PO*_102_. At a ratio of 3:7, *PO*_223_ has 1 pattern ['3|0.0', '5|0.0'] in the PPPs and is related to *PO*_33_, *PO*_102_, and *PO*_143_. At a ratio of 4:6, *PO*_223_ is a part of the training data and is not used in the testing data. As shown in Fig. 1C,D, at the ratios of 1:9 and 2:8, there are no negative observations in training (TRN); therefore, RE and PR are zero. At a ratio of 3:7, an insufficient amount of TRN leads to extreme results; a perfect RE but a relatively low PR are observed. The error rate is less than 0.4 when using a ratio of 7:3. In CMC, profile data are incapable of explaining behavior because (D) indicates that 33% (i.e., (1473 − 980)/1473) of cases are inherently inconsistent.

## Discussion

**Figure 2:**
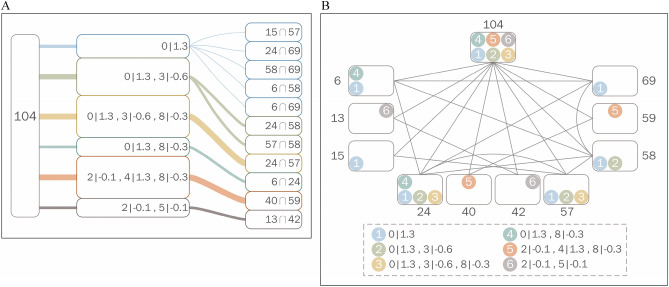
Association between patterns and observations in BCWO (**A**) at the ratio 1:9.

**Figure 3:**
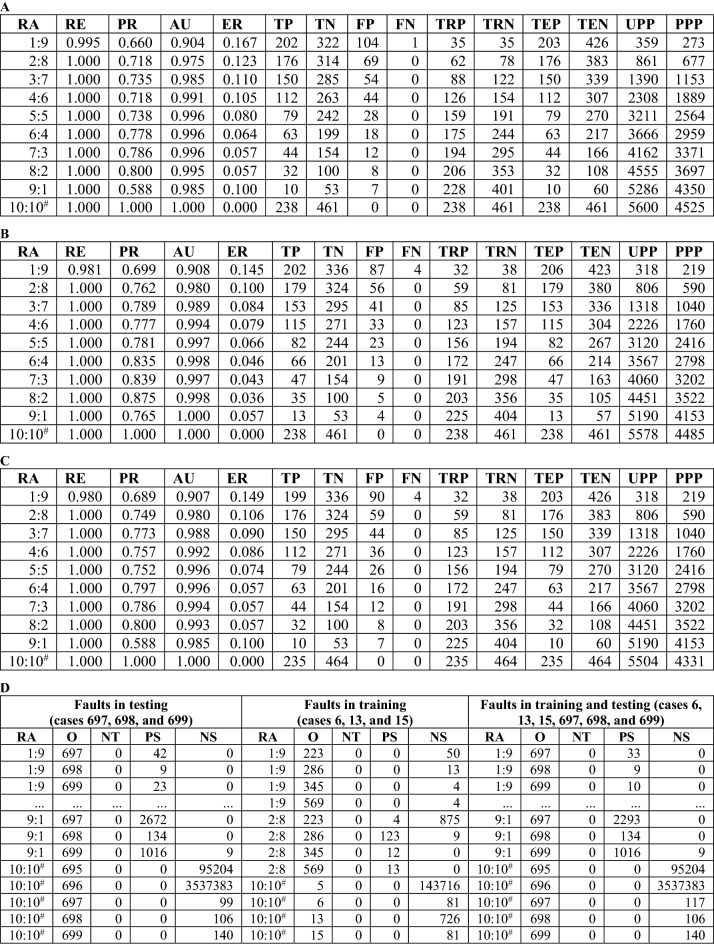
Tolerance to faulty class labels in BCWO (**B**). (**A**) For the case of testing, we change class labels of observations (i.e., 697, 698, and 699) from “1” to “0”. (**B**) For the case of training, class labels of observations (i.e., 6, 13, and 15) are changed from “1” to “0”. (**C**) For both cases, we change class labels of observations (i.e., 6, 13, 15, 697, 698, and 699) from “1” to “0”. (**D**) Although faults in testing, TC can still classify *O*_697_, *O*_698_, and *O*_699_ as PO since the ratio 1:9. Regarding faults in training, the change results in an additional three errors at the ratio 1:9, i.e., *O*_286_, *O*_345_, and *O*_569_. Since the ratio 2:8, the three errors are eliminated due to increased training data. Although faults in testing and training, TC can still classify *O*_697_, *O*_698_, and *O*_699_ as PO since the ratio 1:9. Note at the ratio 10:10^#^, *O*_697_, *O*_698_, and *O*_699_ belong to training data so that their PS are zero; nevertheless, we can identify them by NS.

In Fig. 2, we provide a visualization of the TC method. Compared to the provided images, the analysis of the categorical and numerical data have made it difficult to visualize how the causes are related to the results. Figure 2A shows that the TC method successfully predicts that *O*_104_ is positive because it is related to the six pure positive patterns that are obtained from the respective positive observations in training. Moreover, the thickness of lines represents the score and the degree of positiveness. Figure 2B shows the association between *O*_104_ and other observations. In the group containing *O*_104_, the most common positive pattern is ‘0|1.3’, and *O*_24_ and *O*_57_ have a significant influence on *O*_104_. Figure 3A–C illustrates that the TC method is capable of addressing data with faulty class labels (ground truth) in terms of testing, training, and both testing and training. Figure 3D shows that the TC method can be utilized to correct errors in class labels. Specifically, it is observed that faulty class labels have extreme values of PS or NS, which becomes significant with an increasing amount of data. Based on the profound knowledge of most cases, the TC method could be useful for checking whether the original judgment (e.g., diagnosis) deserves further inspection.

**Figure 4:**
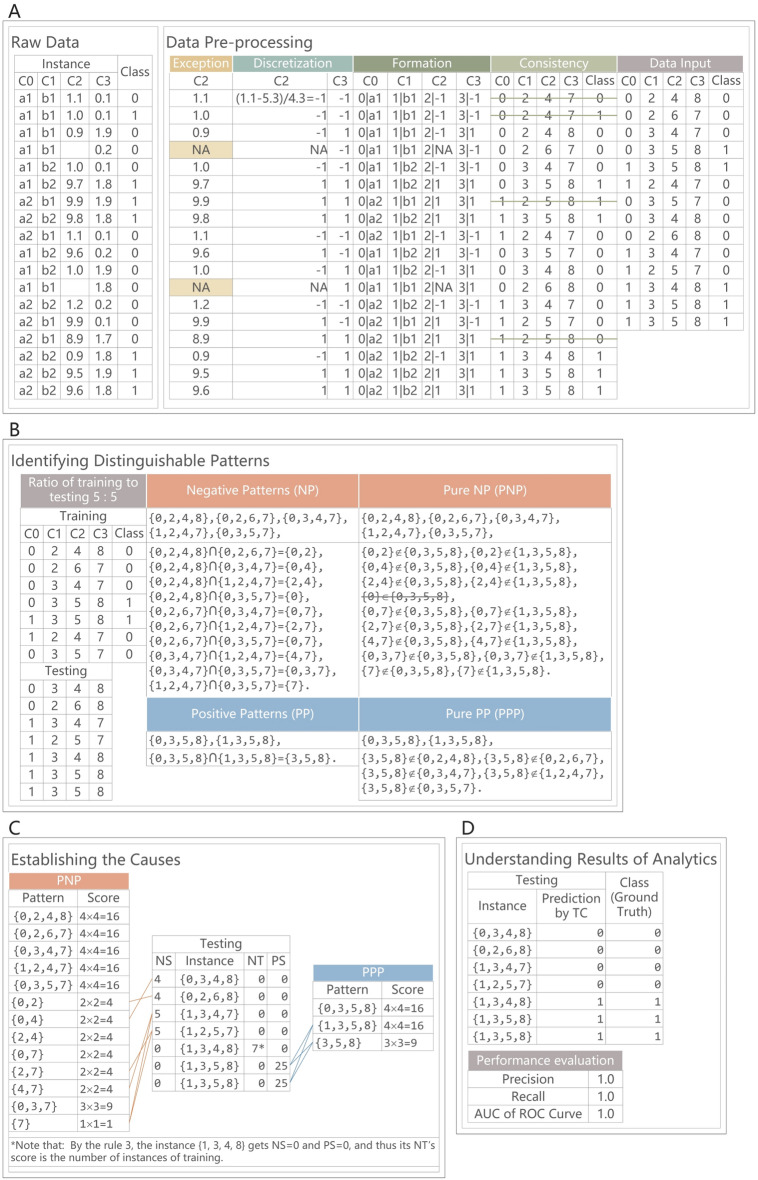
Illustration of TC: a step-by-step approach.

In Fig. 4, a training data to testing data ratio of 5:5 is utilized so that the observations from input data #1 to #7 are used for training a model that is composed of pure positive patterns (PPPs) and pure negative patterns (PNPs). Next, the TC method utilizes the model to predict the class of a test observation. For example, for observation #8, the scores are obtained (i.e., NS = 4, PS = 0 and NT = 0); hence, the TC method predicts that #8 is negative. In particular, observation #12 is predicted to be positive by Rule 3, which shows that the TC method can be used to identify a novel case. In the area of machine learning, the training data to testing data ratio can show the performance of the proposed method. A method is considered excellent if it is accurate in a case that has a few training data but a large amount of testing data, e.g., a ratio of 2:8. Instead of a choosing a random selection, we select the observations based on their sequence so that the experimental results are reproducible. In addition, the ratios from 1:9 to 10:10^#^ are implemented to provide a comprehensive view of the method. In this type of transparent manner, the TC method can help domain experts deeply understand the data.

According to Lim et al.^4^, CMC has an inherent data quality problem, as the minimum error rate of the state-of-the-art methods is greater than 0.4. Although the minimum error rate of the TC method is 0.39, it has a limited ability to deal with this problem. In the TC method, the function of consistency can be used to identify observations that have identical patterns but different class labels to provide an interpretation of the minimum error rate. In social science data, we usually observe that people have identical profiles; however, their behaviors or decisions are quite different. Hou et al.^15^ surveyed several analysis approaches of social media-based applications, which is useful for deeply exploring new significant factors in the classification task.

## Methods

**Figure 5:**
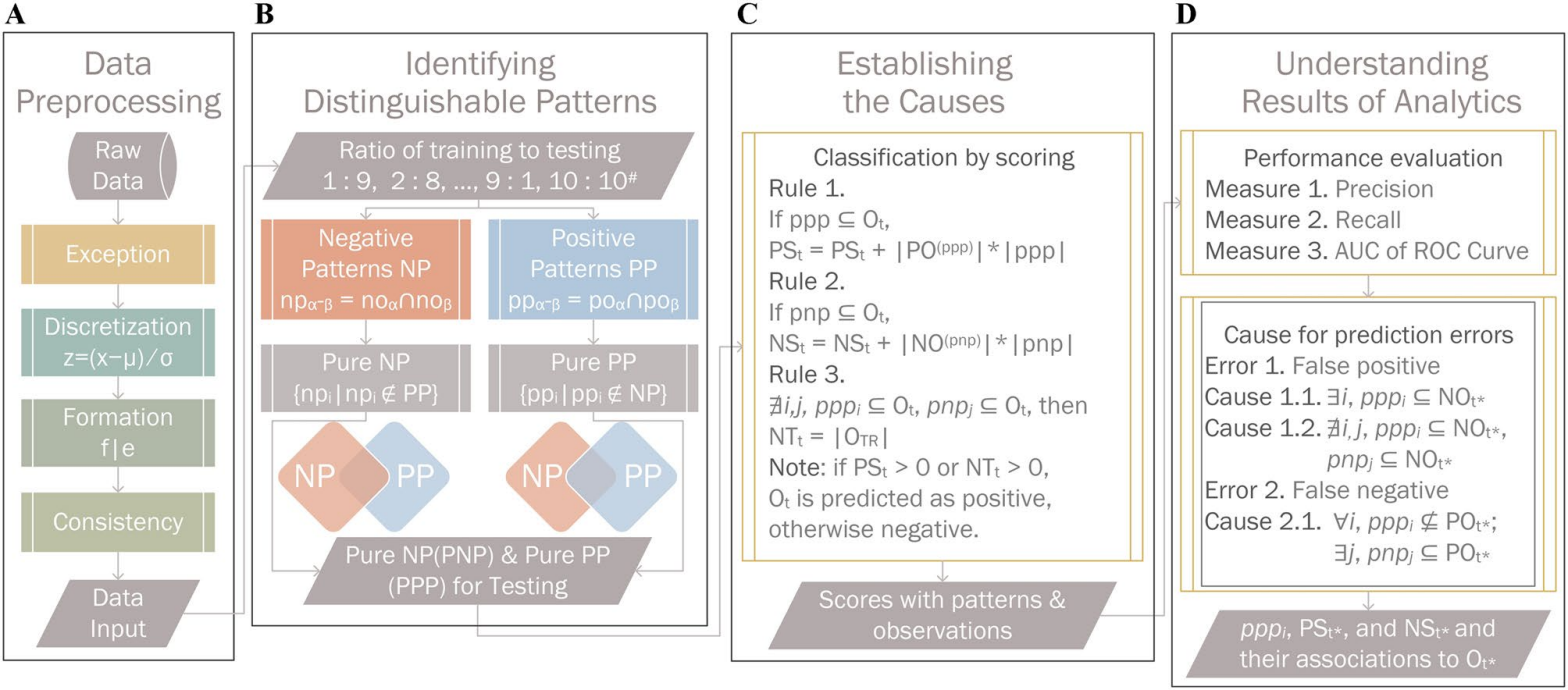
Processes of the transparent classification. (**A**) *Exception* treats missing values as categorical values instead of guesses. *Discretization* transfers numerical values to categorical ones by using z-score, where *x* are numerical values of a feature, *μ* is the mean and *σ* is the standard deviation. *Formation* defines the relations between features *f* and categorical values *e*. *Consistency* removes the contradictory observations that have identical features but different class labels. (**B**) *Ratio of training to testing* divides data into two parts: training and testing. In training, observations are split positive observations PO and negative observations NO. A *positive pattern pp*_*α-β*_ was discovered by intersecting *po*_*α*_ and *po*_*β*_, where *α* = 1, 2, …, *n* and *β* = *α*, *α* + 1, …, *n*, e.g., given *n* = 3, then *pp*_1-1_ = *po*_1_ ∩ *po*_1_ = {*f*_1_|*e*_1,1_, *f*_2_|*e*_2,2_, *f*_3_|*e*_3,1_}, *pp*_1-2_ = *po*_1_ ∩ *po*_2_ = {*f*_1_|*e*_1,1_, *f*_2_|*e*_2,2_, *f*_3_|*e*_3,1_} ∩ {*f*_1_|*e*_1,1_, *f*_2_|*e*_2,1_, *f*_3_|*e*_3,1_} = {*f*_1_|*e*_1,1_, *f*_3_|*e*_3,1_}, *pp*_1-3_ = *po*_1_ ∩ *po*_3_, *pp*_2-2_ = *po*_2_, *pp*_2-3_ = *po*_2_ ∩ *po*_3_, and *pp*_3-3_ = *po*_3_. Note positive observations themselves are positive patterns, e.g., *pp*_1-1_. A *negative pattern np*_*α-β*_ was found by *no*_*α*_ ∩ *no*_*β*_, and negative observations themselves are negative patterns. *Pure PP* (*PPP*) excludes *pp*_*α-β*_ that appears in any negative observation. *Pure NP* (*PNP*) excludes *np*_*α-β*_ that appears in any positive observation. (**C**) In testing of an observation *O*_*t*_, classification by scoring produces five outputs: *PS*, *NS*, *NT*, *PSP*, and *PSO*. *PS* stores observation’s positive score by Rule 1: *O*_*t*_ contains a pure positive pattern *ppp*, increase *PS* by the number of features in *ppp*, multiplied by the number of positive observations containing *ppp*. Rule 2: if *O*_*t*_ contains a pure negative pattern *pnp*, increase *NS* by the number of features in *pnp* multiplied by the number of negative observations containing *pnp*. Rule 3: if *O*_*t*_ does not contain any *ppp* and *pnp*, assign *NT* to the number of training observations. *PSP* stores *ppp*_*α-β*_ related to *O*_*t*_. *PSO* stores the training observations which contain *ppp*_*α-β*_. If *PS* or *NT* is greater than 0, classify *O*_*t*_ as positive otherwise negative. (**D**) *Performance evaluation* demonstrates the accuracy of TC. *Cause for prediction errors*, based on set theory, provides rational explanations for errors caused by TC.

We propose a method of transparent classification, named TC, which not only strives to achieve accuracy but also clarifies the cause of inaccuracy. Furthermore, the design principles of the TC method to ensure reproducibility^11^. Figure 5 shows the processes of the TC method, and Fig. 4 provides a step-by-step approach to implementing the TC algorithms. In terms of *data preprocessing*, missing values and mixed values are addressed. Without randomness and reduction, information on the intrinsic nature of data is provided. In terms of *identifying distinguishable patterns*, patterns are found from training observations, which are used for predicting which class a test observation belongs to, e.g., a malignant or benign tumor. By *increasing the ratios of training to testing observations*, the TC method represents a forest and the trees in the forest, and input data are given in sequence. To avoid CoD, patterns are found by intersecting pairwise observations in each of the classes, which possess essential features of miniature data. In the worst case, only $n \times (n - 1){/}2$ patterns are produced from *n* observations. In contrast, given the challenge of CoD, i.e., given the lowest threshold, *k* items yield 2^*k*^ item sets^12^; this challenge is encountered in KDD, and large amounts of item sets are pruned if the threshold is high. In terms of *positive patterns* (PPs), PPs are obtained from positive training observations (POs). For *pure PPs* (PPPs), any positive pattern that also appears in the negative training observations (NOs) are excluded. By set theory, an exclusion implies where none of the PPPs are included in any of the NOs, and hence the TC method can be used to distinguish between POs and NOs. Analogously, *negative patterns* (NPs) and *pure NPs* (PNPs) are the counterparts of PPs and PPPs. Without fine-tuned parameters or random selection, the uncertainty of the methodology is eliminated. In terms of *establishing the causes*, positive, negative, and novel degrees of a test observation *O*_*t*_ are accumulated by Rules 1, 2, and 3, respectively, which associate patterns with the observation and provide obvious clues for judgment.

1 $${\text{PS}}_{{\text{t}}} = {\text{PS}}_{{\text{t}}} + \left| {{\text{PO}}^{{({\text{ppp}})}} } \right| \times \left| {{\text{ppp}}} \right|{,}\;\;{\text{if}}\;{\text{ppp}} \subseteq {\text{O}}_{{\text{t}}}$$

2 $${\text{NS}}_{{\text{t}}} = {\text{NS}}_{{\text{t}}} + \left| {{\text{NO}}^{{({\text{pnp}})}} } \right| \times \left| {{\text{pnp}}} \right|{,}\;\; {\text{if}}\;{\text{pnp}} \subseteq {\text{O}}_{{\text{t}}}$$

3 $${\text{NT}}_{{\text{t}}} = \left| {{\text{O}}_{{{\text{TR}}}} } \right|{,} \;\;{\text{if}}\;{\nexists }_{{i{,} j}} {,} \;ppp_{i} \subseteq {\text{O}}_{{\text{t}}} {,}\; pnp_{j} \subseteq {\text{O}}_{{\text{t}}}$$

4 $$C{(}O_{t} ) = \left\{ {\begin{array}{*{20}c} {{\text{Positive}},} & {{\text{if}}\;{\text{PS}}_{t} > 0\;{\text{or}}\;{\text{NT}}_{t} > 0} \\ {\text{Negative,}} & {{\text{otherwise}}} \\ \end{array} } \right.$$

In Rule 1, as shown in Formula (1), *O*_*t*_ containing patterns in the PPPs are given a positive score (PS). In Rule 2, as shown in Formula (2), *O*_*t*_ containing patterns in the PNPs are given a negative score (NS). In Rule 3, as shown Formula (3), *O*_*t*_ with no patterns in the PPPs or PNPs is considered novel, and the novelty score (NT) is equal to the number of training observations. The observation *O*_*t*_ is classified, as shown in Formula (4). Regarding notations, *O*_*t*_ is a testing observation, |ppp| is the cardinality of the pure positive pattern ppp, |PO^(ppp)^| is the number of positive observations containing a ppp in the training set, PS_t_ is the positive score for *O*_*t*_, |O_TR_| indicates the number of observations in the training set, and NT_t_ is the novelty score for *O*_*t*_.

To *understand the results of the analytics*, we evaluate the performance of the TC method by three measures: *precision*, *recall*, and the *area under curve (AUC*)^8,12^. According to the standards of diagnostic medicine^13^: AUC = 0.5, no discrimination; 0.7 ≤ AUC < 0.8, acceptable; 0.8 ≤ AUC < 0.9, excellent; and 0 9 ≤ AUC ≤ 1, outstanding. When evaluating the *causes for prediction errors*, error 1 (false-positive^14^), which is denoted as is denoted as *NO*_t*_, occurs if *O*_*t*_ is predicted as positive but is actually negative. In cause 1.1, although *NO*_t*_ contains pure positive patterns, it should not. In cause 1.2, *NO*_t*_ is novel, namely, it has no patterns in the PPPs and PNPs. Error 2 (false negative^14^), which is denoted as *PO*_t*_, occurs if *O*_*t*_ is predicted to be negative but is actually positive. In cause 2.1, *PO*_t*_ contains pure negative patterns but has no pure positive patterns, although it should. Prediction errors occur due to insufficient training data or labeling errors in the training data. Increasing the number of training data helps to reduce prediction errors. If the portion of labeling errors is small, the TC method has the potential to identify labeling errors. Specifically, false negatives usually have a small NS.
